# Evaluation of digital watermarking on subjective speech quality

**DOI:** 10.1038/s41598-021-99811-x

**Published:** 2021-10-12

**Authors:** Yann Kowalczuk, Jan Holub

**Affiliations:** Department of Measurement, Faculty of Electrical Engineering, Czech Technical University in Prague, Technická 2, Prague, 166 27 Czech Republic

**Keywords:** Engineering, Physics

## Abstract

New methods of securing the distribution of audio content have been widely deployed in the last twenty years. Their impact on perceptive quality has, however, only been seldomly the subject of recent extensive research. We review digital speech watermarking state of the art and provide subjective testing of watermarked speech samples. Latest speech watermarking techniques are listed, with their specifics and potential for further development. Their current and possible applications are evaluated. Open-source software designed to embed watermarking patterns in audio files is used to produce a set of samples that satisfies the requirements of modern speech-quality subjective assessments. The patchwork algorithm that is coded in the application is mainly considered in this analysis. Different watermark robustness levels are used, which allow determining the threshold of detection to human listeners. The subjective listening tests are conducted following ITU-T P.800 Recommendation, which precisely defines the conditions and requirements for subjective testing. Further analysis tries to determine the effects of noise and various disturbances on watermarked speech’s perceived quality. A threshold of intelligibility is estimated to allow further openings on speech compression techniques with watermarking. The impact of language or social background is evaluated through an additional experiment involving two groups of listeners. Results show significant robustness of the watermarking implementation, retaining both a reasonable net subjective audio quality and security attributes, despite mild levels of distortion and noise. Extended experiments with Chinese listeners open the door to formulate a hypothesis on perception variations with geographical and social backgrounds.

## Introduction

Watermarking digital medium is a process that has been on the scene of copyright management for 30 years already. Due to quality and user distribution issues, commercial usage of audio watermarking for public distribution has not picked up as expected. However, watermarking in specific markets has revealed attractive potentials, such as communication identification, air traffic control, military or sensible operations requiring security and robustness. Watermarking can be virtually integrated into any digital channel, depending on its contents (audio, video, or text). Thanks to a simple key exchange process, it may be used to trust non-encrypted transmissions, such as telephone or radio transmission; this principle may include emitter identification, using a dedicated watermarking pattern decoded in the receiver. An extension of this principle in encrypted, compressed audio transmissions is of significant interest in modern cybersecurity. Combining watermarking signal patterns with compression algorithms may provide significant advantages in the scope of deploying a transparent security solution.

Therefore, we aim to discriminate the impact of digital watermarking on speech quality when modern techniques are used. Further investigation points to finding the limits of speech that remain intelligible, while watermarking robustness is increased in sacrificing quality. In order to determine these two limits, selected speech samples will be gradually watermarked with increased robustness, leading in progressive speech quality degradation. A primary threshold of quality will be determined and retained as a baseline value for common, public voice exchange. Other effects of noise and environmental disturbances will be added, and the corresponding shift of quality observed and noted. A distortion limit will be estimated, leading to a retained maxima of watermarking robustness that may be potentially used in speech signals. Practically, operational conditions dictate various scenarios containing heavy noise and variable transmission issues. Finally, an attempt to weigh the impact of language characteristics (tonality) and social perception is carried out, replacing the original non-native listeners with Chinese only participants.

Audio quality assessment is regulated by multiple standards. In telecommunication transmission quality tests, the ITU-T P800 Recommendation is widely used. It states conditions for subjective audio quality assessment; the subjects must be seated in an anechoic or semi-anechoic listening environment, with specific guidelines for sample creation and playback.

## Speech watermarking

### Validated models

**Figure 1:**
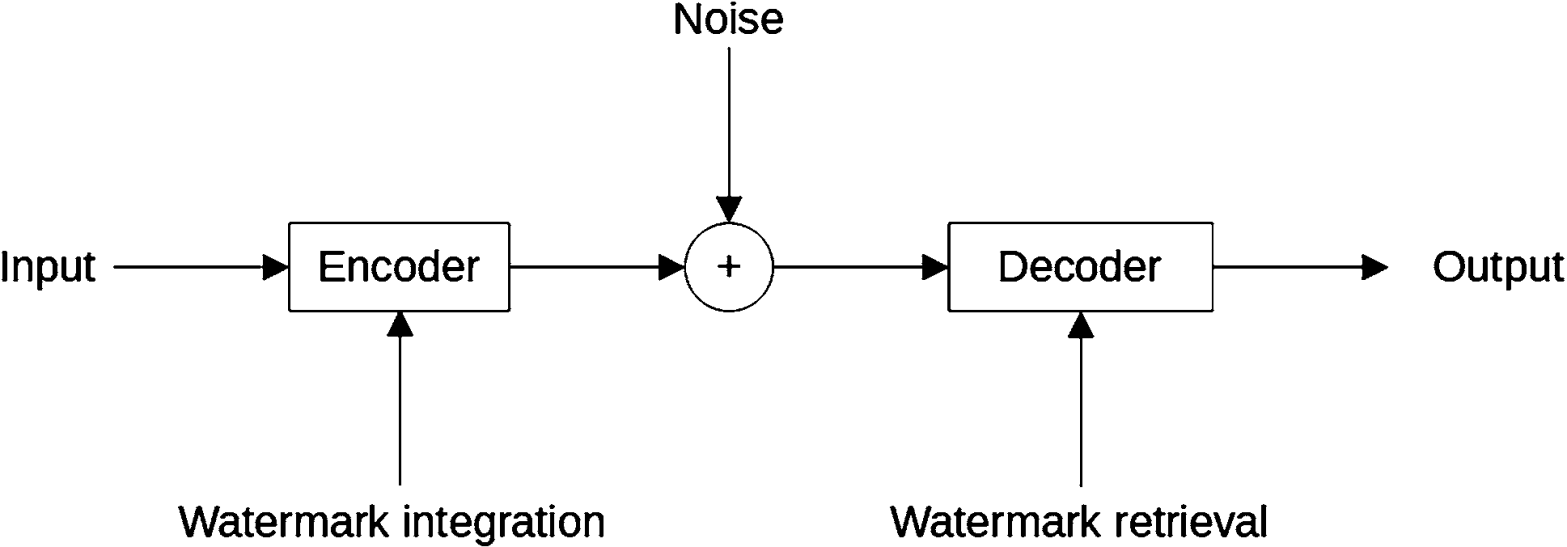
Watermarking principle.

Digital audio watermarking consists of embedding a payload into an audio signal. A recovery process allows authenticating the content as the genuine one. For the transmitted signal being used as the support for the watermark, a compromise has to be set between robustness, distortion/fidelity, data payload inside the watermark, and security level. The principle flow chart of this process is visible in Fig. 1. Data such as a digital message, sometimes referred to as key^1^, that may consist of various forms, is defined as the watermark. Various procedures to embed the watermark inside the original signal further enhances its degree of robustness. A watermark may take different structures: an encrypted or modulated speech content, a pseudo-random sequence, simple pre-defined bit sequences, etc. Consequently, the inputs to watermark generators are highly diverse. Speech watermarking techniques may be additive, multiplicative, or quantized, and the watermarks may be embedded in the time or frequency domain.

Depending on the strategy adopted, some techniques are more robust to some given forms of attacks, with an apparent relationship to the signal quality and the influence of the chosen watermarking method. Watermark detection (or recovery) is classified as being blind or non-blind (also called informed). Informed/non-blind detection requires a copy of the clean, original signal to be available, or at least a fragment of it. Due to its lack of flexibility and higher compromised integrity in the interception, this kind of detection is not considered in our study. Recovery without the passing of the original signal for watermark detection is called blind detection.

As the watermark robustness and perceptibility are competing factors, our goal is to define quality and security thresholds adapted to the targeted category of speech transmission. Further requirements include the survivability of the watermark to distortion factors and noise or the superimposition of both (for example, in military transmissions). Typical metrics^2^ of a watermark’s efficiency are data payload (the number of watermark bits per unit of time) and security (the level of stiffness against removal, embedding, or detection). Additionally, and especially in a mobile environment, real-time operation requirements may be a factor limiting the design of heavy or complex methods involving too high a demand in computational power. Audio domain processing requires the watermark to be as transparent as possible or for the common ear to remain imperceptible. Thanks to good knowledge and modeling of the human perception of speech, it is possible to approach those demands efficiently. Further, on the side of quality^2^, it is essential to differentiate fidelity from signal quality. An audio sample quality might be poor even before watermarking, but the output watermarked signal can be of solid fidelity. Watermarking process must not affect audio fidelity beyond a defined set of standards. Significant speech properties defining perception levels include consonants, short-term spectrum, spectral slope, formants number, location, bandwidth, and amplitude.

Speech watermarks may be embedded in the time or frequency domain of the signal. Most algorithms are based either on time, Discrete Cosine Transform (DCT), Fast Fourier Transform (FFT), or Discrete Wavelet Transform (DWT). An early and basic way to input audio watermarks may be provided by modifying the amplitude of the speech in the time domain. Another time-domain technique aims at replacing the Least Significant Bits (LSB) of the original signal by the watermark, with a low effect on perceptibility. The higher the payload, the more bits are used to embed additional data. Some forms of attacks may predictively look for such a concatenation. Therefore its robustness is judged somewhat fragile. Further, more complex time-based strategies introduce the watermark as a shift-delayed echo of the original signal. There may be significant gains in robustness by choosing pseudo-coding schemes that emulate noise patterns while perceptibility is kept at a low level. Alternatively, and recognized as a more sophisticated approach, speech watermarks can be created by modifying the magnitude or phase coefficients in the DCT, FFT, or wavelet coefficients.

Modern algorithms^3^ are taking advantage of research in human speech models, which implies an alteration of signal properties that are not directly related to the audio samples. Such approaches largely enhance the coherence of the watermark to the speech and provide much higher degrees of reliability. Among others, and exhaustively described^2^, this may include Linear Prediction (LP) coefficients, Log Area Ratio (LAR), Inverse Sine (IS) coefficients, Line Spectrum Pair (LSP) parameters, and Reflection (PARCOR) coefficients. Further mentioned properties include the auto-correlation coefficients and the cepstral coefficients. As written previously, watermarking algorithms directly integrating such models present a sensitive and efficient way to solve the sophisticated task of making the process as transparent as possible. A revolutionary concept of watermarking called frequency masking provided significant improvements in the trade-off between robustness and perceptibility. The effect of frequency on the human ear is not linear^2^, but logarithmic. Speech loudness is not evenly perceived in its entire frequency range. A minimum intensity level defines the threshold of sensitivity of the human ear at a specific frequency. Loud audio waves are known to hide or “mask” adjacent, low-level sounds. If multiple audio signals are received in a limited frequency window, the louder sound will mask the weaker signal, leading to frequency masking. The masking level is dependent on the frequency, intensity level, and the nature of the audio signals reaching the listener.

Audio watermarking techniques are extensively reviewed in recent publications, summarizing their specific mechanisms and physical principles^4^. Due to the variety and originality of each method and their adaptation, it is advisable to use available comparison means and carefully select the type of watermarking algorithm that best suits the project needs.

### Recent techniques and research

Further frequency domain watermarking is credited with a very extensive research^5^. To take advantage of various methods’ benefits, novel research now incorporates multiple frequency-transform processes, so-called hybrid-transform. Multiple methods have been challenged to achieve better results by merging two or more watermarking variants. An example of such a hybrid method, Direct Cosine Transform–Direct Wavelet Transform (DCT–DWT), reveals that DWT is an excellent technique to achieve robustness and that DCT reduces the original signal’s distortion. A combination of more contents is even possible^6^, which also emphasizes the usage of the DCT–DWT method. The spatial-frequency distribution properties of DWT enhance the robustness of the watermark, and DCT offers energy spreading in the low-frequency spectrum, which makes it desirable for compression methods, an essential chapter of digital media nowadays.

The mainstream of innovation concerns image watermarking; however, those experiments are continuously transitioned to audio and textual contents. Recent watermarking techniques now systematically include some subjective quality evaluation, which has become a concern in modern telecommunication matters. Due to the absence of a common benchmarking evaluation standard for watermarking performance, useful research^4^ reviews the most recent and typical watermarking schemes available on the market explicitly.

### Language and social characteristics

There is a significant difference in the definition of languages from populations around the world^7^. On a perceptive basis, especially when analyzing subjective quality in transmissions, we distinguish tonal languages (Chinese Mandarin) from intonation languages (English). The tone and pitch variations of tonal languages have lexical and grammatical implications that may alter the whole meaning. For a native speaker of a tonal language, a foreign language that is intonation defined may well imply a difference of perceived quality^8^.

A further fixed bias to consider is the social perception and characterization of quality. Cultural background may play a role in the way that foreign voters may perceive speech quality. Depending on the environment they are used to evolve in, the quality scale that is defined for a given parameter may well have a definition bias depending on the voter’s background^9^. Past analysis and standard’s definition from ETSI^10^ have already noticed significant and repetitive bias when comparing results between Mandarin listeners and other non-native subjects (mainly of European backgrounds). A systematic higher score was observed throughout different experimental conditions.

## Methods

### Samples preparation

Preselected, available speech samples published by ETSI were chosen. The targeted objective was the watermarking impact on the audio quality; potential influencing variables were kept as neutral as possible. Samples’ length and gender voices are normalized at 4 seconds, with male and female speakers alternatively recorded, allowing for favorable post-statistical analysis. Audiowmark, an open-source software developed by Stefan Westerfeld^11^, was used for embedding watermarks in these samples. It is a command-line application that allows to read a chosen sound file and store a 128-bit message (defined as a key in the documentation) in the output file. Audiowmark uses the patchwork algorithm to embed the watermark in the spectrum of the input file.

Patchwork by itself is the idea of incorporating minor changes to the original signal or adding a certain amount of measured and limited variation to it. Initially derived from image watermarking progresses^6^, the values that reflect the changes in the signal shall correlate with the watermark strength. It is applied to a limited, small segment of the host audio selected randomly that gets added with a specific statistic (for example, Gaussian distribution).

Once the average values of the clean and the modified samples are computed, their difference is calculated and used to determine if the watermark is indeed present. The latest evolutions of this method usually involve a dual-channel statistical approach based on pseudo-random processes^12^. We may adequately define the modified signal as distorted since some of its contents are intrinsically altered. Accordingly, this reflects one of the facts being researched in our experiment, the quantitative perceptible distortion and its correlation to the watermark encoded strength. Encryption characteristics of watermarking keys have not explicitly been evaluated or tested. Therefore the notion of key length remains a topic for additional research. Furthermore, the content of that key itself shall not be mistaken with the notion of the watermarking message (sometimes ambiguously referred to as “key”) that is embedded in the transmitted signal. Our present research is focused on speech quality subjective perception, we do not include here further investigation on the cryptography or validity of the key complexity, but we retain those points as a further branch of investigation and evaluation. By setting the strength parameter of the watermark, we may induce more or less distortion on the host signal, increasing its robustness at the same time. In order to determine a threshold of perceptibility, a variety of watermark strength has to be challenged to human ears, along with the degree of recovery of the watermark itself.

Technically with Audiowmark, the audio signal is split into 1024 sample frames. After the computation of their FFT coefficients, the frames’ amplitude spectrum is altered with pseudo-randomly selected values. Those slight variations of amplitudes serve as a base for watermark detection post-treatment. The algorithm used here is inspired by Martin Steinebach, in his thesis “Digitale Wasserzeichen für Audiodaten”. The patchwork algorithm is analyzed in detail in available documentation and publications^1^.

### Practical testing

A wide range of testing conditions was selected, allowing for the versatility of observations inside one experiment. Three different noise conditions that reflect real-life environments encountered daily were then added. Several watermarking degrees were inserted into different recording conditions, mainly:

- Original studio recording with clean voice and quiet environment. Watermarked strength with values of 10, 30, 75, 200, and 650.
- Simulated engine noise from HMMWV transport vehicle, with a 3 dB Signal to Noise Ratio. Added watermark with strength of 10 and 30.
- Simulated restaurant/pub noise with a 6 dB Signal to Noise Ratio. Watermark strength also set at 10 and 30.
- Acoustic recording with mild effects such as reverb, and mixed variably with previous noise. Here watermark strength was spread at 30, 100, and 500.

Subjective testing methodology is following ITU-T Recommendation P.800. Guidelines include testing environment specifications, acoustic tuning of playback equipment, and calibration values of sound pressure, allowing for a correct approach of statistical comparison. 12 samples per listening condition were compiled for a final selection of 16 listening conditions. A panel of subjects was invited to evaluate the listening quality of those 192 samples using a dedicated professional voting system. A total of 8 votes per sample was chosen to be representative and obtain detailed statistical data.

The 16 specific conditions are described below:

- C01: Studio recording, clean reference sample.
- C02: Studio recording, watermarked strength 10.
- C03: Acoustic recording of original studio sample.
- C04: Studio recording, watermarked strength 30.
- C05: Pub noise, added in the background of studio sample.
- C06: Pub noise, watermarked strength 10.
- C07: Studio recording, watermarked strength 75.
- C08: HMMWV tactical vehicle noise, added in the background of original studio sample, watermarked strength 10.
- C09: HMMWV tactical vehicle noise, added in the background of original studio sample.
- C10: Pub noise, watermarked strength 30.
- C11: Studio recording, watermarked strength 200.
- C12: HMMWV tactical vehicle noise, added in background of original studio sample, watermarked strength 30.
- C13: Pub noise, recorded acoustically, watermarked strength 30.
- C14: Studio recording, watermarked strength 650.
- C15: Studio recording, recorded acoustically, watermarked strength 500.
- C16: Studio recording, recorded acoustically, watermarked strength 100.

Subjects were selected as a mixed group of non-native listeners (Central European and expatriated citizens), with almost equally allocated gender repartition. This was a voluntary choice to minimize any statistical deviations from language dependencies or gender influence (both being the theme of further experiments). Voters have been familiarized with the voting procedure before the sessions. The setup was kept as simple as possible, and the voting population widened to achieve the highest amount of statistical results. Age distribution was recorded between 20 and 50 years old for all participants. The group consisted of 16 subjects, with a gender split of 8 males and 8 females.

The second experiment involving Chinese listeners focused on native mandarin speakers, which were temporarly or permanently expatriated and familiar with the English language. Gender and age selection were similar to the previous population, and the group consisted of 24 subjects, with a gender split of 11 males and 13 females. Individual listening order and voting were recorded, and results are analyzed in the next section.

## Results

The distribution of votes is sorted for conditions and averaged to compare the initial, clean studio recording. The votes are based on a MOS (Mean Opinion Score) scale, as described by ITU-T Recommendation P.800. The scores are described in Table 1.

**Table 1:** MOS score quality equivalence.

MOS score	Corresponding quality
5	Excellent
4	Good
3	Fair
2	Poor
1	Bad

Reference speech samples shall match a score located around 5 or 4.5 for narrow-band recordings covering the 300 Hz–3.5 kHz spectra, while heavily distorted or unintelligible speech quality scores shall be leveled around 1.

**Figure 2:**
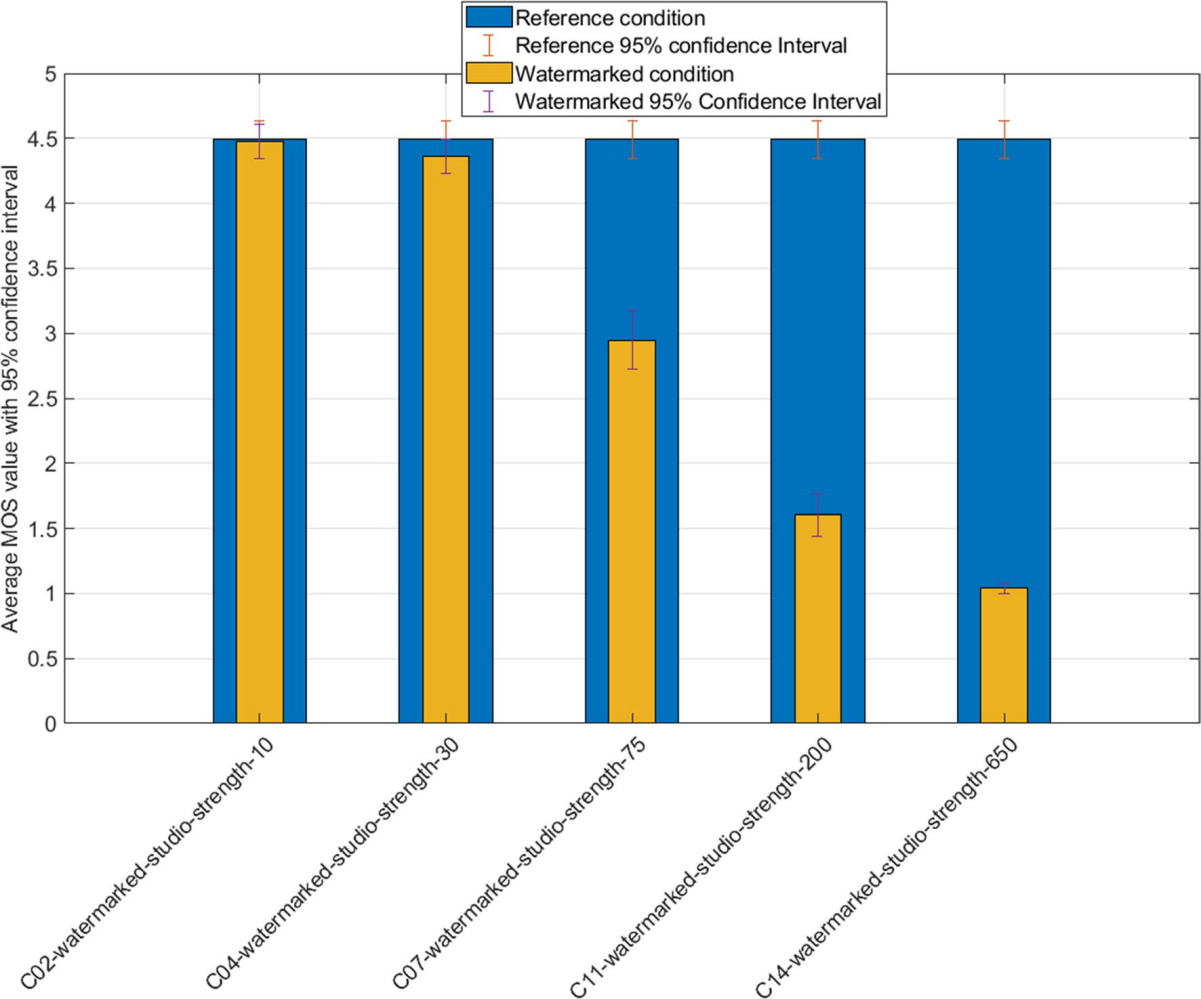
Results of reference studio condition with increasing watermark strength.

Figure 2 compares studio recordings with increasing watermarking strengths. We obtain an even distribution of the scores, with a noticeable degradation of the speech quality happening only at a fairly high level of watermarking (strength 75 and above). All watermarks could be retrieved even at the lowest strength settings.

In order to determine the statistical differences between our results, we use Student’s Dependent Groups t-test, single-sided at 95% confidence level. The calculated values are shown in Table 2, following Fig. 2 analysis.

**Table 2:** t-Test: results of reference studio condition with increasing watermark strength.

Condition	Reference condition	t-value
C02	C01	0.104
C04	C01	1.243
C07	C01	11.402*
C11	C01	25.928*
C14	C01	44.882*

Statistically important differences ($\alpha =0.05$ critical value 1.662) are marked with *character.

We remark noticeable differences starting with a watermark strength of 75 and above, while the T-value increases proportionally with the strength parameter. This reveals a two-fold outcome following those values:

- A watermark strength of up to 30 does not produce a significant degradation of perceived speech quality in these specifically mentioned conditions. It means that strength coding impact on quality will first be noticeable with a strength level between 30 and 75.
- Perceived quality significantly and quickly degrades above strength values of 75, until reaching a stable minimum MOS of 1 (practically inaudible speech).
- Despite moderate to heavy strength settings, perceived quality keeps decent values given the amount of distortion introduced.

**Figure 3:**
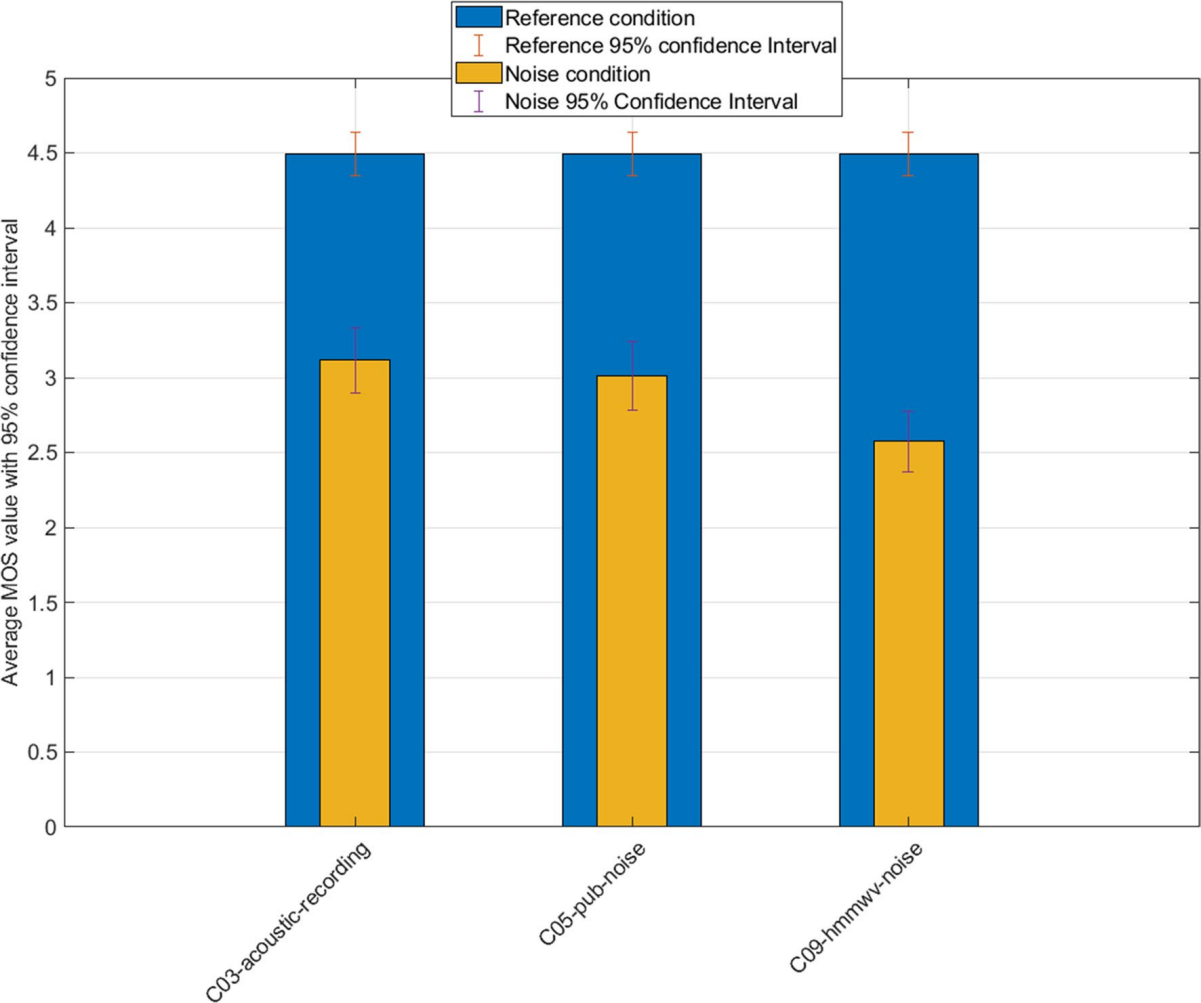
Results of reference studio condition and noise conditions without watermark.

For noise influence visualization, we next plot on Fig. 3 the scores between the clean studio sample and the corresponding sample with added noise. Noise introduction significantly lowers the initial quality of speech, which was expected. The voters still ticked mostly fair scores, with slight variations depending on the type of disturbance employed. We obtain a relatively even distribution of the votes despite the differences in noise introduced.

**Figure 4:**
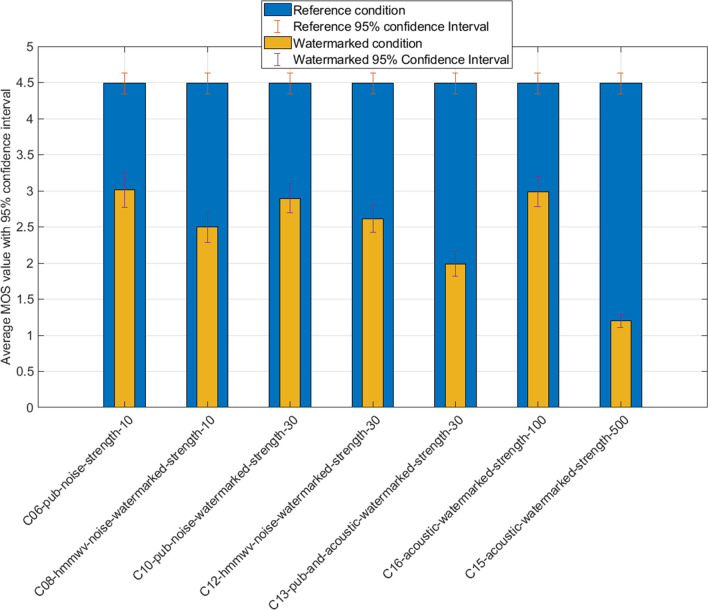
Results of reference studio condition with noise and increasing watermarking strength.

We introduce in Fig. 4 the watermarked sample with embedded noise and notice that we obtain a quite uniform distribution of the scores. This can be interpreted as the low perceptibility of the watermark distortion on speech, compared to the actual noise of the condition.

As we can see, the most severe effect is experienced with acoustic recording. It may be explained by the speech envelope being downgraded (primarily by multiple reflections) and the corresponding dynamic lowered, adding to the noise effect. Again, watermarks could be retrieved despite the noise. This is in line with the assumption that the slight speech distortion will remain mostly unnoticed in average or higher noise conditions, making it suitable for transmissions of such nature.

**Figure 5:**
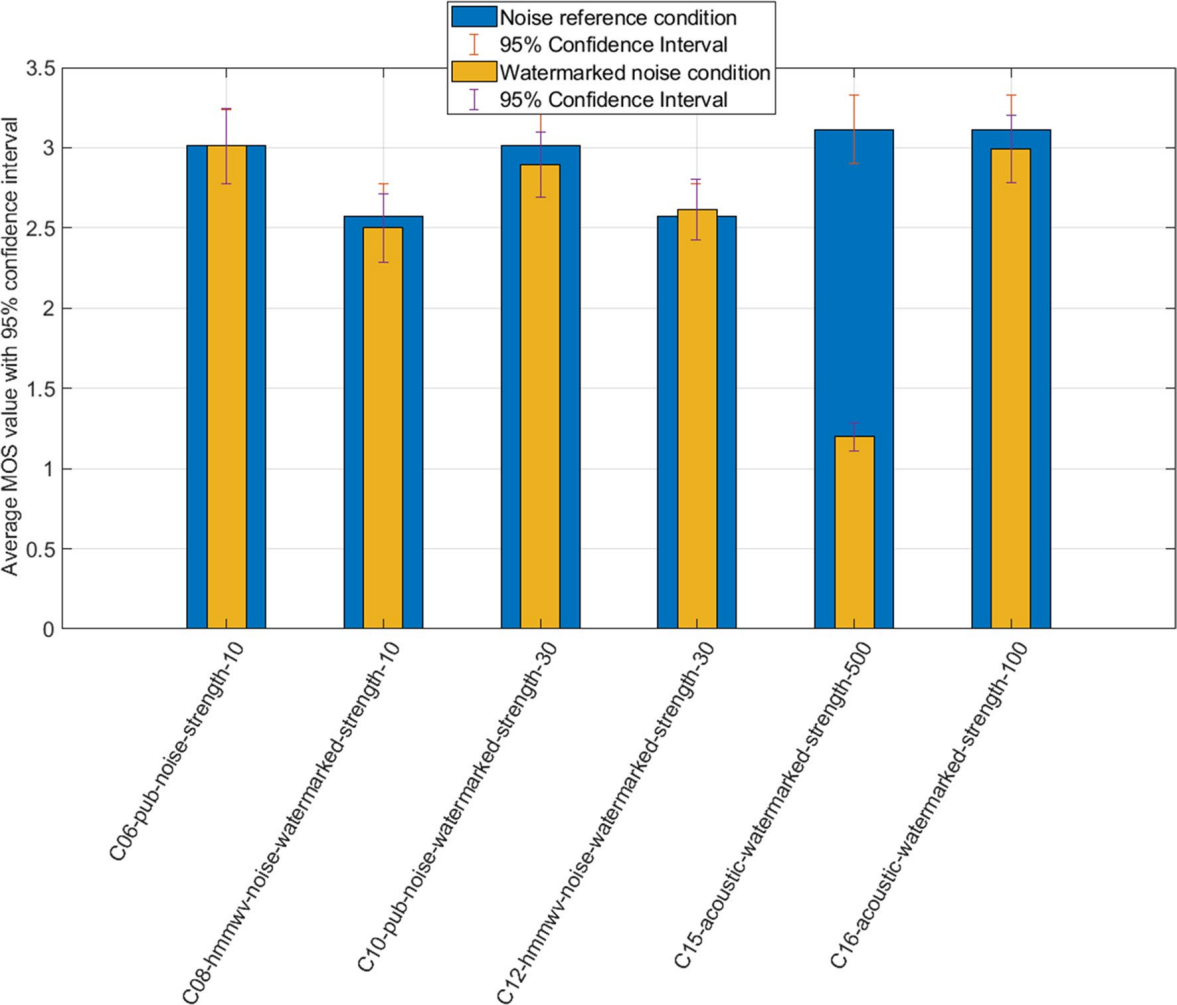
Results of reference noise condition and increasing watermarking strength.

The previous hypothesis may be confirmed by plotting in Fig. 5 the samples containing the background noise as a reference and the vote results of the same noisy samples with increasing watermark strength. We repeat the statistical evaluation with Fig. 5, in Table 3.

We can notice a breaking difference in the distribution of statistical values in those conditions, mainly:

- A rather uniformly distributed difference between the original noise condition and its watermarked version (except for the extremely heavy strength value of 500).
- Most significantly, watermarking of light to moderate strength has no impact on the perceived speech quality.
- Watermark-induced distortion seems to be “lost in the noise” for human hears, up to some critical strength numbers (post 100 in our case).

We see clearly that reasonably watermarked speech in a noisy environment remains in an acceptable “fair” quality range. Very high watermarked samples are not of acceptable quality; however, mild-strength values between 30 and 75 lead to a good compromise in noisy environments.

Studio samples constitute well-suited candidates for watermarked Signal to Noise Ratio evaluation. The absence of background noise may give an objective, physical way to measure the impact of watermarking on the original speech. A future study may determine if a degree of correlation exists between the subjective speech quality scores and a given range of SNR values. As multiple parameters interact inside our samples, a precise protocol will need to be defined for such an investigation.

**Table 3:** t-Test table: results of reference noise condition and increasing watermarking strength.

Condition	Reference condition	t-value
C06	C05	0.000
C08	C09	0.488
C10	C05	0.738
C12	C09	0.293
C15	C03	16.117*
C16	C03	0.815

Statistically important differences ($\alpha =0.05$ critical value 1.662) are marked with * character.

**Figure 6:**
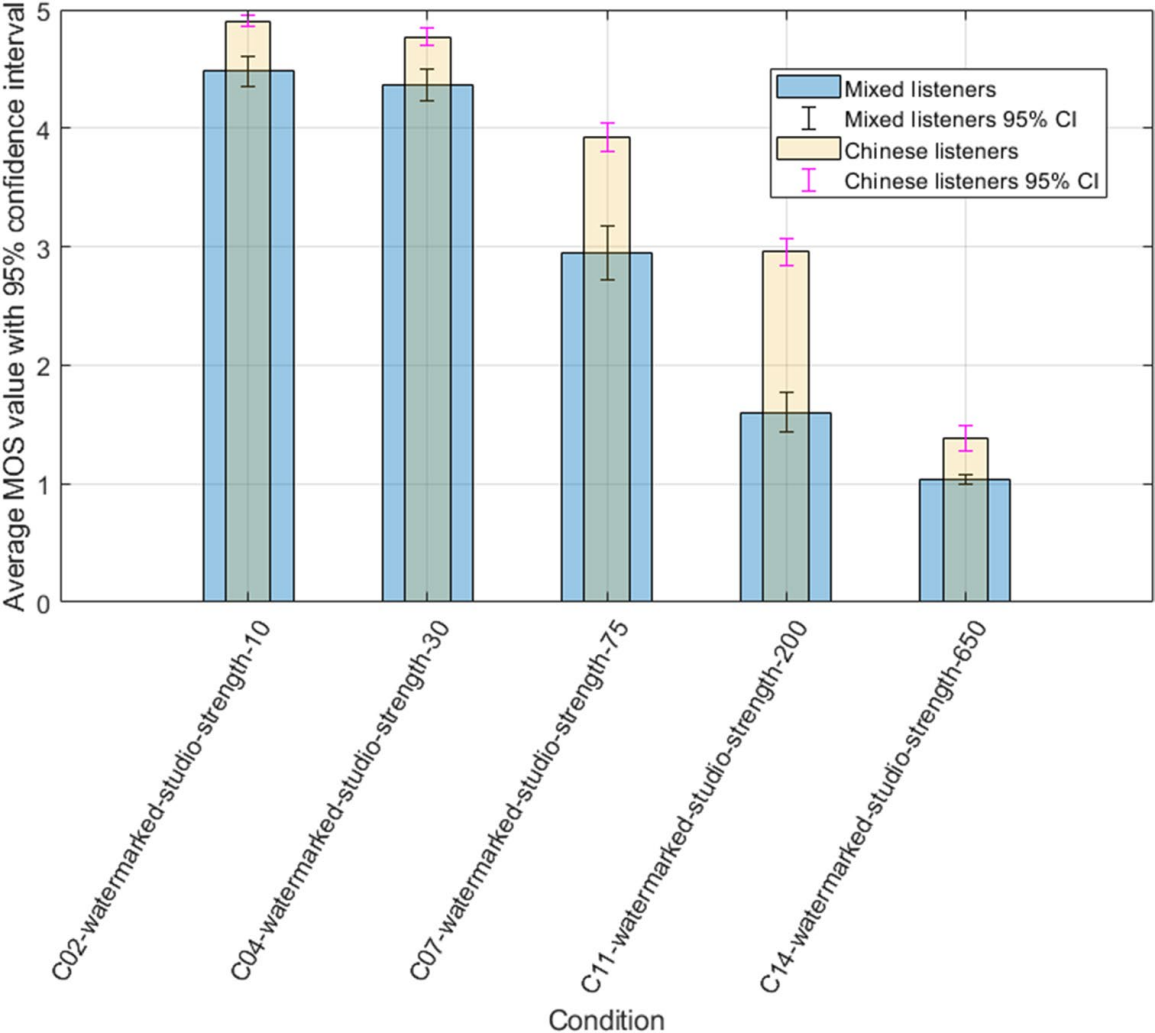
Comparison of studio conditions between mixed and Chinese listeners.

**Figure 7:**
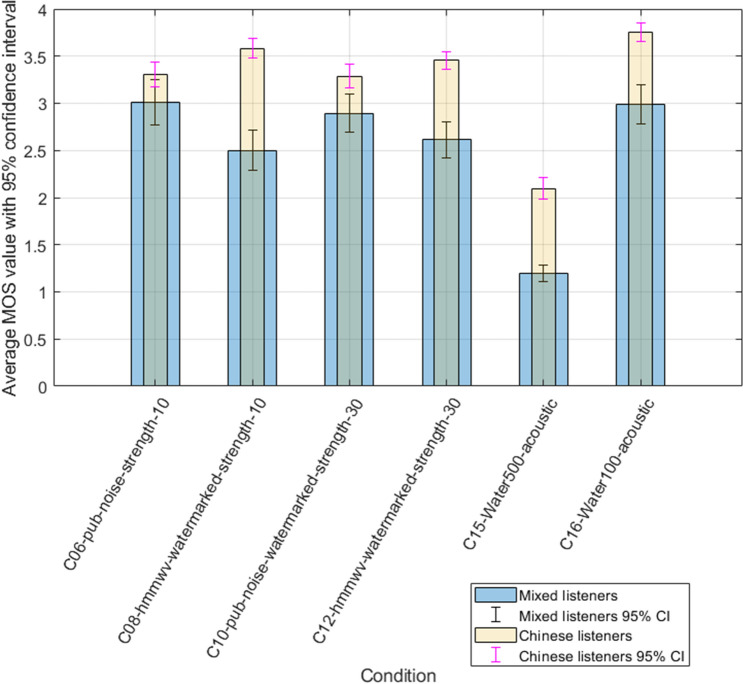
Comparison of watermarked noise conditions between mixed and Chinese listeners.

Our last experiment challenges the social and geographical background of listeners with the global results voted. We compare the original panel of non-native listeners (Central European auditors) with Chinese only listeners. Figure 6 shows the differences observed between both groups with the studio only conditions, whereas Fig. 7 gives an overview of noisy watermarked conditions.

We can identify a positive difference/bias between both groups of listeners, with the results of the Chinese group being systematically more optimistic. Independently of noise or watermarking distortion, Chinese listeners repeatedly produce a better score than the mixed group. The variation ranges from 0,3 up to 1,5 points on the MOS scale. That difference seems to be specially marked when sample conditions are degraded, and intense noise is present. Those results are in line with ETSI experiments involving Mandarin listeners^10^, which revealed a systematic positive bias ranging from 0. 5 to 1.5 MOS. Due to the nature of subjective testing itself, enhanced data collection and careful analysis could help formulate a more precise explanation of those findings. Human and psychological cross-analysis (including psycho-acoustic mapping) might help establish a detailed explanation of these results. A further investigation involving different tonal languages and cultural backgrounds may allow the formulation of additional hypotheses linked to those findings. Our current results do not provide the possibility of identifying the reason involved in this difference; careful evaluation of different listeners with restrictive language and social selection could provide additional insights.

## Conclusion

As reviewed in this experimental research and subjective testing, watermarked speech samples using the patchwork algorithm show that this technique is robust and may be retrieved at low watermark strength, even in noisy conditions. The induced acoustic signal alteration and distortion allowed us to go beyond the initial analysis of the watermarking technique and showed encouraging results in terms of perception in noisy environments. Further development of the initial setup led us to compare listeners from specific geographical origins (Chinese citizens only), with unexpectedly positively shifted results. Overall, this research was the opportunity to observe and reunite the interaction of human factors with transmission technologies and encryption, opening various hypotheses that could be challenged in further experiments.

## Discussion

The review of speech watermarking influence on subjective perception with a commonly available algorithm resulted in invaluable observations. Statistically, independent voting results reveal that light to moderate levels of watermarking robustness do not affect the perceived speech quality. Simulating more realistic daylife environments, moderate to solid watermarks remain lost in noise for the average listener. Our perceptibility threshold is located at a watermark strength ranging from 50 to 75, while values up to 100 seem acceptable in terms of recognized speech quality. The watermark may be retrieved in very challenging noise conditions at those strength levels and opens the door to further experiments with high distortion and low-bitrate compression testing.

Proper scaling of the watermarked distortion shall be determined by further testing, as SNR values alone may provide guidance but not a direct representation of the actual modification of the host signal. Selected research introduced a notion of “Signal to Watermark Ratio,” which might be a viable metric for further scaling. As the experiment deliberately limited the acoustic bandwidth between 300 Hz and 3.5 kHz, about typical speech transmission frequencies, a targeted “audiophile” setup, involving high-quality musical samples only, could reveal fundamental differences in the obtained results. In order to eliminate additional potential noise in the statistical analysis, the listeners’ acoustic sensitivity could be challenged before the listening tests by a simple mapping of their audio capacity between 20 Hz and 20 kHz. The results obtained under heavy noise conditions show that speech perception remains correct while introducing a subtle distortion. Therefore, further experiments may be relevant when voice and noise are directly recorded and injected on the transmission medium with the watermark embedded.

Our final social experiment resulted in statistically significant findings, suggesting that language type or cultural background could influence the perception of quality of the voters. A systematic positive bias has been observed with Chinese listeners, which opens the door to further research with different languages or geographical origins. This could imply that an adaptation of the MOS scale would be possible depending on the listener’s background or that even the actual perception of quality may vary with the origin of the voter. Those results converge into the idea that geographical and sociological background might influence the way humans perceive speech and potentially various forms of audio signals. It could constitute a novel approach to the definition of perceptive quality and play a role in human factors’ analysis. Various additional experiments could bring additional data and help enhance those hypotheses, such as comparing gender perception of speech or repeating such setups in different parts of the world with both native and non-native listeners.
